# Retraction: Molecular separation of ibuprofen and 4-isobutylacetophenone using octanol organic solution by porous polymeric membranes

**DOI:** 10.1371/journal.pone.0350712

**Published:** 2026-06-02

**Authors:** The PLOS One Editors

The *PLOS One* Editors retract this article [1] due to concerns about compromised peer review and compliance with PLOS policy on Manipulation of the Publication Process.

All authors either did not respond directly or could not be reached.
